# Criminal Lunatics
**Temple Bar.* No. I.*Criminal Lunatics*, a Letter to the Chairman of the Commissioners in Lunacy, by W. Charles Hood, M.D., Physician to Bethlehem Royal Hospital. London. chill. 1860.


**Published:** 1861-01

**Authors:** 


### ART. III.—CRIMINAL LUNATICS.*

AN attempt to popularize the subject of Criminal Lunatics, which appeared in the New Serial of last month, and which is founded upon, and indeed is mainly a reproduction of, Dr. Hood's letter to the Chairman of the Commissioners in Lunacy, renders it in some sort incumbent on us to examine the views put forth in that pamphlet—a task which, from a cursory perusal of the publication when first it appeared, we did not then feel called upon to undertake.

Dr. Hood's exponent in our able contemporary, *Temple Bar,* adopts (by implication, at least) all that gentleman's views and suggestions for the amendment of the law relating to Criminal Lunatics. These views we shall proceed to consider *seriatim* and in order.

Our author in introducing his subject, remarks that the term "criminal lunatic" is at present indiscriminately employed in relation to two distinct classes of offenders—namely, those who having been exempted from judicial punishment on the ground

---

* *Temple Bar.* No. I.
*Criminal Lunatics,* a Letter to the Chairman of the Commissioners in Lunacy, by W. Charles Hood, M.D., Physician to Bethlehem Royal Hospital. London. chill. 1860.

of insanity, are thereupon ordered to be detained in safe custody during her Majesty's pleasure, and those who have become of unsound mind while undergoing punishment for offences committed when sane, and who ought therefore to be termed "*insane convicts*," or "*insane prisoners;*" and he afterwards states that it is to the mode in which the first class, those acquitted on the ground of insanity, ought in future to be treated, that he wishes chiefly to solicit attention. There is a slight inaccuracy involved in treating the first class, as including only those acquitted on the ground of insanity; since it also includes those who, being found insane upon arraignment, are not called upon to plead, and, therefore, not being tried, cannot be said to be acquitted. This is an oversight of little importance, apart from the evidence it affords of the imperfect state of the writer's knowledge of his subject. It is impossible, however, to read without astonishment and some degree of alarm an assertion made by the Physician of the Bethlehem Royal Hospital, that the return of sanity in no sense implies or necessitates the restoration to liberty of a person confined under a warrant for "safe custody during her Majesty's pleasure." We apprehend, and we shall continue of this opinion until we have reason to know that the officers of State act upon any other hypothesis, that, excepting cases in which the original acquittal is deemed to have amounted to a miscarriage of justice, the return of perfect sanity being proved, the prisoner's discharge would be allowed as a matter of right. Having regard to the present imperfect state of mental science, the Legislature has thought fit in the interest of the community, but not by way of punishment, to place the liberty of criminal lunatics at the absolute disposal of the Executive; but this power was granted and is held upon an implied trust, that liberty should be granted when the public safety no longer required that it should be withheld; and if this trust were repudiated by the Executive, as it seems to be by our author, Parliament would find it necessary to provide for the public safety in some other way. The lack of knowledge, indeed, is the only justification for this expedient; and whenever philosophers or psychologists are able to demonstrate the truth of any general rules whereby we may know that a man once proved lunatic has recovered the perfect use of his faculties, the Executive will assuredly be called upon to resign this extraordinary power into judicial hands. Dr. Hood proposes that, as regards lunatics guilty of slight offences, "namely, those convicted of assault, want of securities, and such like,"* the juris-

* In the previous paragraph we are informed that "it is the privilege of every English subject to be held responsible to the laws of his country only so long as he remains of sound mind," the writer ought therefore to know that the persons he is writing about cannot be convicted.

diction of the Commissioners in Lunacy should be enlarged, and that they should be empowered to discharge such prisoners on their restoration to reason, on the guarantee of relations or friends that they should be prevented from again disturbing the public peace. Our knowledge of law does not justify us in making any observations upon this proposal, so far as it relates to the case of those unhappy individuals, if any such there be, who are "convicted of want of securities;" but with regard to lunatics "convicted of assault," in so far as the suggestion rests upon the idea that this is a slight offence, a little consideration, we think, will show that it is inadmissible. An assault committed by a lunatic is a totally different thing from an assault committed by a person whose impulses are under the control of reason; in the one case, the act is a crime the magnitude of which is fairly measured by the injury done, because it can be properly assumed that the offender intended to do what he did, but no more. In the other case the act is no crime, the malicious intent being wanting; but the controlling power being also wanting, it is impossible, in the abstract, to assign any limit to the danger to be apprehended from an individual who may have committed any breach of the peace in a state of lunacy. We do not say that every lunatic who commits a breach of the peace ought to be confined for life; but we say, that the propriety of liberating such a person involves considerations perfectly independent of the nature of the specific act which may have brought him within the cognizance of the law.

The question still remains whether, under any circumstances or with regard to any class of cases, the power of discharging persons under confinement for "safe custody during her Majesty's pleasure," could be properly transferred from the high officers of State with whom it now rests to the Commissioners in Lunacy. We think it could not. The power in question should be exercised judicially, following upon an inquiry as to whether the applicant for liberty has been restored to reason, or to such a degree of reason that he may with safety to the public be admitted to liberty, conditional or unconditional, according to the circumstances of each particular case.

In reference to this inquiry, it is the duty of the Commissioners in Lunacy to watch over the interests of the public which are necessarily opposed to those of the individual; and the performance of this duty is therefore incompatible with the exercise of the judicial function of deciding the question raised by the inquiry.

Dr. Hood considers that some legislative measure is required, which should define under what circumstances, and to what extent, if at all, persons coming under the following classes may be discharged :—

"*Firstly*, those who, under the influence of mental disease, have committed or attempted murder or other grave offences, and who are liable to such sudden ebullition of homicidal mania, that no laws can bind them, and no companion or friend be safe from their attack; and, *secondly*, those who have feigned insanity to escape conviction, and who, through the exertions of astute counsel, supported by *ex-parte* medical evidence, have been acquitted of murder or attempted murder, or other grave offences, on the ground of insanity, but who, nevertheless, through a series of months or years, while subject to the close surveillance of an asylum, evince no symptom of a disordered mind." (p. 7.)

Dr. Hood himself offers no suggestion as to the nature of the measure which he thinks is required; nor does he define the circumstances which, in his opinion, would justify the discharge of either of these classes. At this omission we are not much surprised, as with respect to persons coming under the first class we suspect it would tax his ingenuity not a little to suggest any possible circumstances which would justify their discharge at all; and with respect to the second, the question is how are we to get rid of such a class altogether. Can we by any improvement in the system of criminal procedure greatly lessen or altogether prevent the failure of justice which the existence of such a class denotes?

It would appear from the next suggestion made by Dr. Hood, two pages after the last quotation, that this question was not absent from his mind, though he has not placed it clearly before his readers. His suggestion is that—

"Whenever insanity is *set up* as a defence, the trial of the prisoner for the offence with which he is charged, whatever may be the stage at which it has arrived, should be postponed until the state of the prisoner's mind at the time has been made the subject of inquiry, at the same or some following session or assize, as the case may be, and as then shall be directed before an ordinary jury, whose verdict of insanity, if such it shall be, shall in all cases be recorded by the Court, as an answer to the previous indictment. It seems, indeed, [says Dr. Hood] altogether inconsistent and contradictory to allow a prisoner to allege that he did not commit the offence for which he is charged, and that when he did commit it he was of unsound mind; yet both these grounds of defence are admissible under the plea of not guilty." (p. 10.)

The miscarriages of justice to which reference has been made are mainly due, as we have before stated in this Journal, to the necessarily imperfect opportunities which the witnesses to the state of the prisoner's mind have had of forming the conclusions upon which their evidence is based; and we have already pointed out that a simple remedy for this evil would be found in giving to the judge in criminal cases, the power which he has in civil cases, of adjourning a trial after it has begun, for the purpose of obtaining further evidence. Under ordinary circumstances, no doubt such a power is not needed on the criminal side; because, unless the prosecutor makes out his case, the accused is entitled to his discharge; and unless he is able to prove his accusation, he ought not to bring the case into court: but where the act charged is proved, and the defence relied on is insanity, provided there be some evidence of it, there appears to be no objection to a reasonable delay, for the purpose of further evidence if necessary. This is a defence which the accused is bound to prove affirmatively as satisfactorily, as the prosecutor is bound to prove the facts charged in the indictment; and if successful, the prisoner is not entitled to his discharge from custody: therefore if the point at issue is in any degree doubtful, no question regarding the liberty of the subject stands in the way of the course proposed. So much, therefore, of Dr. Hood's suggestion as relates to the postponement of trials where evidence of insanity is offered in defence, coincides with views which we have previously expressed, and which we still retain; but for the rest we entirely dissent from him. We think that so much of the indictment as depends upon the facts alleged, should be proved and found by the jury before any adjournment should be ordered; and as to its being inconsistent, and contradictory, to allow a prisoner to allege that he did not commit the offence, and that when he did commit it he was insane, the supposed inconsistency and contradiction are pure figments of the writer's own invention, since it is quite unnecessary that the prisoner should allege that he did not commit the offence. The prosecutor alleges that he did, and is bound to prove it. Every legal authority agrees, that the plea of not guilty amounts to no more than the mere expression of a wish to be tried according to law, and is not an assertion of innocence.

A further suggestion which Dr. Hood thinks it necessary to make for the amendment of the law is thus expressed:—

"Again: it seems proper that some statutory provision should be made whereby those prisoners who become insane before trial, or are found upon arraignment to be of unsound mind, might, upon recovery, be duly tried and disposed of according to law, without leaving the responsibility of advising prolonged imprisonment or immediate discharge to the Secretary of State for the Home Department." (p. 12.)

Fortunately for the credit of the law, but most mal-apropos to his suggestion, Dr. Hood quotes the following case, which he thinks "will *perhaps* show the necessity for some such enactment."

"D. D. was indicted for murder at the Worcester assizes, 1852, and being found insane at the time of arraignment, was without delay removed to a lunatic asylum. In the summer of 1856, having recovered from the mental disease, he claimed to be tried for the offence with which he was charged, and being found guilty of manslaughter, was sentenced to two years' imprisonment. This term of punishment he underwent, and at the end of the period was discharged." (p. 12.)

What D. D. did under the law as it stands, his prosecutor might have done, and therefore the suggested amendment is unneeded. In a subsequent page, however, Dr. Hood, assuming the duty of a parliamentary draughtsman, gives us his views of the required amendments more precisely in the shape of clauses to be inserted in an Act of Parliament to be passed, repealing the existing statutes relating to criminal lunatics, and re-enacting the valuable parts of each with Dr. Hood's new clauses. Referring to this part of his pamphlet, we gain a fresh insight as to the views with which he makes the last suggestion we have quoted, and as to the bearing of the cases of J. F., J. P., and C. B. W., as quoted in support of such suggestion. It appears, then, as part of Dr. Hood's scheme for the amendment of the law, that, on a prisoner pleading insanity, the truth of that plea is to be tried before trying the truth of the facts charged in the indictment; that, if the defence is proved, the prisoner is to be sent to an asylum, whence, upon its being certified that he has become of sound mind, he is to be brought to trial upon the original indictment, and if found guilty—which he may be, *notwithstanding he was previously found insane at the time of committing the offence,* then "the Court shall record the sentence that would have been passed if the prisoner had been found guilty of the offence, and not insane at the time of committing it." (p. 23.)

Pausing here, let us consider how this would work in the case of a lunatic charged with murder. He is brought to trial, and pleads insanity at the time of the offence. The trial is thereupon postponed, and another jury is empanelled at a subsequent court to try the alleged insanity, and if they find for the prisoner he is sent to an asylum, where it is to be hoped there will be so little attention paid to his disease that he will not recover; for if he do, it will be the duty of the surgeons attending him to certify the fact to the Secretary of State, whereupon the unhappy man will be again brought to trial, and if found guilty, as he most likely will be, of the offence, but insane at the time of committing it, the Court will record sentence of death—that is to say, the sentence that would have been passed if the prisoner had been found guilty of the offence, and not insane at the time of committing it. Dr. Hood, however, seems to have reconsidered this possible event after going to press; for in a foot-note on page 24 he tells us that "prisoners of this kind found guilty of murder, or any offence for which the sentence of death or imprisonment for life would have been passed, will remain in confinement in a prison, asylum, or other proper receptacle for life."

We gather from this that before the Act goes into the Queen's printer's hands, a clause will be added to this effect. Great care should be taken that this is not overlooked; for as the text stands at present, the poor man will inevitably be hanged for an act committed when admittedly insane.

It is but fair to add, however, that it is only in the serious cases that the lot of the criminal lunatic will be so frightfully altered by this new law; for if the sentence be one of imprisonment for a definite term, then it will be taken to have commenced when he was first sent to an asylum; and if the time he has been there is equal to the term of his sentence, he will get his immediate discharge; if not, he will only have to make up the difference. Any way, however, after this Act passes, we must all understand, that short of death, lunatics will have to submit to the same punishments as other men. To render the law consistent, however, with itself, it will be necessary to pass another short Act, declaring that from the date of the passing thereof, malicious purpose, or felonious or unlawful intent, shall not be required to be proved in order to establish any criminal offence, any law, statute, or custom to the contrary thereof in anywise notwithstanding.

---

